# Graft Selection in Anterior Cruciate Ligament Reconstruction: A Comprehensive Review of Current Trends

**DOI:** 10.3390/medicina60122090

**Published:** 2024-12-20

**Authors:** Marko Ostojic, Pier Francesco Indelli, Bruno Lovrekovic, Jerome Volcarenghi, Doria Juric, Hassan Tarek Hakam, Mikhail Salzmann, Nikolai Ramadanov, Aleksandra Królikowska, Roland Becker, Robert Prill

**Affiliations:** 1 Sports Traumatology Division, Traumatology Department Draskoviceva, University Hospital “Sestre Milosrdnice”, 10000 Zagreb, Croatia; marko.ostojic@ymail.com; 2 Osteon Orthopedics and Sports Medicine Clinic, 88000 Mostar, Bosnia and Herzegovina; 3 Südtiroler Sanitätsbetrieb, 39042 Brixen, Italy; pindelli@stanford.edu; 4 Institute of Biomechanics, Paracelsus Medical University (PMU), 5020 Salzburg, Austria; 5 Department of Orthopaedics, University Hospital Merkur, 10000 Zagreb, Croatia; blovrekovic5@gmail.com; 6 Department of Orthopaedic Surgery and Traumatology, Centre Hospitalier Universitaire Helora, Site Kennedy, 7000 Mons, Belgium; jerome.valcarenghi@gmail.com; 7 Department of Orthopaedics, University Hospital Basel, 4031 Basel, Switzerland; doria.j@hotmail.com; 8 Center of Orthopaedics and Traumatology, Brandenburg Medical School, University Hospital Brandenburg an der Havel, 14770 Brandenburg an der Havel, Germany; hassanhakam151995@hotmail.com (H.T.H.); m.salzmann@uk-brandenburg.de (M.S.); nikolai.ramadanov@gmail.com (N.R.); roland_becker@yahoo.de (R.B.); 9 Faculty of Health Science Brandenburg, Brandenburg Medical School Theodor Fontane, 14770 Brandenburg an der Havel, Germany; 10 Physiotherapy Research Laboratory, University Centre od Physiotherapy and Rehabilitation, Faculty of Physiotherapy, Wroclaw Medical University, 50-367 Wroclaw, Poland

**Keywords:** anterior cruciate ligament injury, anterior cruciate ligament reconstruction, graft selection, individualized approach, bone patellar tendon autograft, quadriceps tendon autograft, hamstrings autograft

## Abstract

Anterior cruciate ligament (ACL) injuries are common in sports and often require surgical intervention, e.g., ACL reconstruction (ACLR), aimed at restoring knee stability and enabling a return to pre-injury activity levels. The choice of graft is crucial, impacting biomechanical properties, clinical outcomes, and complication rates, and is especially important in revision surgeries after graft failure. Over the past 30 years, trends in graft selection have evolved towards more individualized approaches, considering factors such as patient activity level, prior injuries, and tissue availability. In Europe, autografts like hamstring tendon (HT), bone-patellar tendon-bone (BTB), and quadriceps tendon (QT) are preferred, with the increasing use of QT grafts. This review synthesizes the current literature on graft selection and its influence on ACLR outcomes.

## 1. Introduction

Anterior cruciate ligament (ACL) injuries are a significant challenge in sports medicine, frequently requiring surgical intervention to achieve optimal recovery and return to pre-injury activity levels. ACL reconstruction (ACLR) is one of the most common orthopedic procedures, with an incidence of 74.6 per 100,000 people annually [1]. The primary goal of ACLR is to restore knee stability; however, outcomes can vary, with re-rupture rates ranging from 2% to 40%, depending on the patient’s activity level and specific demands [2,3,4,5]. For elite athletes, this injury can result in 9–12 months away from competition, underscoring the critical importance of every aspect of the surgical process [6]. Among the many decisions in ACLR, the choice of graft type stands out as one of the most crucial and modifiable factors [7,8]. This decision becomes even more vital in revision surgeries, where graft failure can be career-ending for athletes in cutting sports and a devastating personal experience [9]. Over the past three decades, graft selection has evolved significantly, reflecting deeper insights into tissue biology and individualized patient care. Understanding the history and evolution of ACLR brings a more thorough understanding of the procedure itself [10]. ACLR in the 21st century should be performed in an individualized manner, focusing particular attention on tissue biology—no single graft is suitable for all patients [11]. The level of activity of the patient, as well as the patients’ life plans and their profession, should be considered. The preference of surgeons and the skill level required for each specific procedure are of utmost importance. For example, an expeditious and efficient graft harvest can make the procedure quicker and reduce the risk of complications. Prior injuries and tissue availability are non-changeable factors and should be prioritized in evaluating the graft choice. Grafts are routinely classified as autografts, which are those taken from the patient, and allografts, which are those obtained from donors. Autografts, including hamstring tendon (HT), bone-patellar tendon-bone (BTB), and quadriceps tendon (QT), are overwhelmingly preferred for primary ACL reconstruction (ACLR) compared to allografts in Europe [12]. However, another option is the usage of the peroneus longus split graft, as recently described [13]. The BTB autograft used to be the golden standard until the end of the 1990’s, followed by the usage of HT autografts and QT autografts, which seems to have become more popular recently. [10]. In the last 20 years, many graft choices have emerged, with some being used for the first time, like the plantaris autograft [14]. This review explores the shifting trends in graft choice, analyzing the recent literature on the biomechanical properties, clinical outcomes, and complications associated with different grafts. Understanding the history and current practices in graft selection provides a comprehensive view of this essential aspect of ACL reconstruction. The double bundle technique was designed to more accurately replicate the natural anatomy of the anterior cruciate ligament by reconstructing both its anteromedial (AM) and posterolateral (PL) bundles.

## 2. Graft Selection

Graft selection should be personalized, considering factors such as gender, age, profession, activity level, and specific needs, including occupational demands. This review provides a comprehensive analysis of the most commonly utilized graft options, offering insights into their respective advantages and considerations.

### 2.1. Bone-Patellar Tendon-Bone Autograft

The BTB autograft has long been considered the gold standard for ACLR due to its superior biomechanical properties and the effective healing of the graft–tunnel interface, characterized by bone-to-bone healing [11]. First introduced by Jones in 1963, the BTB graft quickly gained widespread acceptance due to its favorable clinical outcomes by the standards of its time [15]. By the late 1980s and early 1990s, it had become the most widely utilized graft in ACL reconstruction, particularly among athletes seeking a return to high-demand, cutting sports activities.

The BTB graft is harvested from the central portion of the patellar tendon, typically measuring 10 mm in width, with bone blocks attached at each end from the patella and tibial tuberosity. This configuration offers several biomechanical advantages. The bone-to-bone healing process is not only faster but also stronger than soft tissue healing, providing more rigid fixation strength, which is particularly important in the early stages post-surgery [16,17]. The inclusion of bone blocks also allows for press-fit fixation, which has demonstrated improved outcomes, including lower graft failure and revision rates [18]. Additionally, the ligamentization process of the intraarticular portion of the graft progresses more rapidly with BTB grafts compared to hamstring tendon (HT) grafts [19,20]. On static laxity testing, the BTB graft is stronger compared to hamstring tendon autografts [21]. The tunnels used with BTB grafts can be round, conical (on the femoral side), or rectangular [22,23,24]. However, the clinical relevance of the shape of the tunnel remains questionable. A comparative in vivo study using dynamic biplanar radiography found no statistically significant difference in postoperative anterior tibial translation (ATT) between HT and BTB grafts in ACL reconstruction [25]. Due to the two bone plugs, fast incorporation of the graft can be expected. A CT based study has shown almost complete bony integration 8 weeks after surgery. In contrast, it takes significantly longer when soft tissue grafts are used.

Numerous studies have shown that patients undergoing ACL reconstruction with a BTB graft achieve favorable clinical outcomes, including high rates of graft survival, low rates of re-rupture, and a high percentage of patients returning to their pre-injury activity levels [26]. Two recent systematic reviews highlighted BTB and QT grafts as excellent choices for skeletally immature patients (Tanner stages 3 and 4) in terms of functional outcomes and stability [27,28]. When considering revision rates, BTB grafts continue to produce the most favorable results among all graft choices. For instance, the Norwegian registry reports a 2-year revision rate of 1.2% for BTB grafts, compared to 3.6% for quadriceps tendon (QT) and 2.5% for HT grafts [2], a finding that has been confirmed by an all-Scandinavian registry [29]. Studies indicate that, regarding failure rates, BTB grafts are superior to HT grafts [30] and offer comparable results to QT grafts [31]. It has also been shown that BTB grafts result in faster Return to Play (RTP) and lower failure rates compared to HT grafts [6]. However, no significant differences in patient-reported outcome measures (PROMs) were found between BTB and HT autografts in long-term, multicenter randomized controlled trials [32], nor between BTB and QT grafts [33]. Regarding maturation time, both in terms of morphologic (arthroscopic findings) and MRI parameters, BTB grafts have shown superiority over HT grafts [34]. An isokinetic study revealed no difference in extensor strength between the BTB and HT groups, though the BTB group demonstrated higher flexor strength [35]. While a return to sport rates was similar across graft types, BTB grafts showed a tendency for a better return to pre-injury sporting activity levels compared to HT grafts [36]. In contrast, allografts did not show promising results in terms of return to sports when compared to BTB grafts [37].

However, the use of BTB grafts is not without drawbacks, which are primarily related to donor site morbidity. Anterior knee pain and pain with kneeling are common complications associated with BTB grafts, often due to harvesting of the patellar tendon [38]. These issues can be particularly problematic for athletes engaged in activities that require frequent kneeling or jumping. Additionally, harvesting the BTB graft can lead to other complications at the donor site, such as patellar fractures, extensor tendon rupture, patellar tendinopathy, and patella infera, potentially affecting both rehabilitation and long-term knee function. The development of minimally invasive harvesting techniques has mitigated some of these complications, potentially leading to a resurgence in BTB use in modern ACLR [39]. However, BTB grafts still result in more pronounced kneeling pain, graft site pain, and sensitivity loss compared to QT grafts [33]. Another potential drawback is graft–tunnel mismatch. Recent studies have also reported that cyclops lesions are more common with BTB grafts than with HT grafts [40]. Additionally, there is evidence suggesting that BTB grafts may carry a higher risk of subsequently developing knee osteoarthritis (OA) compared to HT grafts [41,42]. Beside the fact that the BTB graft is, from the biomechanical point of view, a very strong graft, there is a potential risk of over tension of the graft causing reduction in rotation or ROM [43].

### 2.2. Hamstring Tendon Autograft

Hamstrings play a crucial role in maintaining knee stability, particularly by supporting the anterior cruciate ligament (ACL) and counteracting anterior tibial translation. The hamstring’s muscles are ACL agonists, generating posterior shear forces, especially when the knee is partially flexed, which helps to stabilize the knee by pulling the tibia backward. Additionally, they counteract valgus forces and external tibial rotation, positions where ACL injuries are most likely to occur [44,45]. As already mentioned, the BTB autograft has traditionally been regarded as the “gold standard” for ACL reconstruction, but the HT autograft has gained significant popularity among orthopedic surgeons worldwide. Typically, the semitendinosus tendon is harvested and prepared, in general, as a quadruple graft. Sometimes it is used in combination with the gracilis tendon in cases where there is a lack of an appropriate graft diameter or graft length. Graft diameters of less than 8 mm seem to show a higher failure rate according to the Danish registry [46].

Fixation techniques vary, with direct methods like bio-interference screws minimizing longitudinal and transverse forces on the graft, while indirect methods, such as suspensory button fixation, offer ease of application and improved graft-to-bone contact but are prone to the “bungee effect”, causing complications like tunnel widening [7,47]. Although some research indicates that suspensory fixation can loosen and lengthen under cyclic loading, this occurrence has not been correlated with adverse clinical outcomes. Further research is needed to fully understand the biomechanics behind unsuccessful ACL reconstruction outcomes, particularly regarding tunnel positioning and graft fixation [48]. Specifically, tunnel positioning seems to be one of the most common reasons for failure after ACL reconstruction.

Traditionally, the HT autograft has been associated with lower rates of anterior knee pain, minimal donor-site morbidity, ease of harvest, satisfactory patient-reported and functional outcomes, and preservation of the extensor mechanism [9,38,48,49]. Moreover, ACL reconstruction using an HT autograft has proven successful even in older patients, allowing them to return to their desired levels of activity [49].

Given the impact of osteoarthritis (OA) and its prevalence in the ACL-reconstructed population, it is essential to examine the factors contributing to its development, particularly in the context of allograft usage. Understanding these factors could aid in designing specific rehabilitation protocols tailored to the type of graft used in each patient. Research has shown that patients with HT autografts have a similar prevalence of osteoarthritis as those with other autografts such as QT [41]. Multiple mechanisms may influence OA development following ACL reconstruction with an HT autograft. Residual anterior tibial translation, which can persist even after ACL reconstruction, may over-constraint the patellofemoral joint, leading to patellofemoral joint (PFJ) arthritis [44,50]. Additionally, altered knee kinematics, such as increased internal tibial excursion and decreased knee flexion, can trigger osteoarthritic changes in the knee following ACLR [50]. While much research focuses on knee extensor strength, deficits in knee flexion strength after ACLR with an HT autograft should not be overlooked, as they can impact knee function and contribute to OA development [45,50].

Beyond potential functional deficits, one of the most significant concerns following ACLR is reinjury and the potential need for revision surgery, particularly in athletes aiming to return to their pre-injury sports participation. Research by Bloch et al. suggests that decreased hamstring strength and reduced semitendinosus pre-landing activity after ACLR with HT autografts may play a critical role in knee reinjuries [51]. While maximal muscle strength is important, explosive muscle strength should also be emphasized as a protective factor against reinjury, as ACL injuries typically occur within 50 ms of foot-to-ground contact. Studies have shown that explosive hamstring strength recovers more slowly than maximal strength following ACLR with hamstring tendons, with significant hypotrophy and strength deficits in knee flexors persisting for extended periods. These deficits can create an unfavorable biomechanical environment in the knee, resulting in suboptimal predictors of reinjury risk, such as limb symmetry index and hamstring-to-quadriceps maximal torque ratio [45,52,53]. These functional deficits, coupled with the time required for graft maturation, have led to the recommendation of a minimum nine-month return-to-sport (RTS) period following ACLR with an HT autograft [45,54]. However, muscle function and coordination capability seem to be the key for the patients in terms of RTS and the actual time should be based on an individual decision.

One of the challenges associated with using hamstring tendons in ACLR is the unpredictability of graft diameter. Both patient-related and surgical factors can result in a suboptimal graft size. Research suggests that HT autograft diameters of 8 mm could reduce unfavorable clinical outcomes, since it has been shown that grafts above 8 mm have higher load-to-failure and lower risk of revision with better functional outcomes than the ones below this figure. In addition, a 0.8-fold decrease in revision rates has been noted with every 0.5 mm increase in graft diameter after the 7 mm threshold [55]. Although some research has proposed various methods for increasing the graft diameter such as the addition of allografts or folding a graft to make it five-stranded, no techniques has been shown to be successful enough for it to become routine practice. On the contrary, the addition of an allograft has been correlated with a higher risk of revision surgery because of graft incorporation interruption. Moreover, attention should be directed to other important factors proven to have a direct impact on the surgical outcomes, such as adequate care of menisci, correct placement of anatomical tunnels, and bone morphology including tibial slope and intercondylar notch [56,57]. Recent research suggests that it is possible to predict HT graft dimensions based on a patient’s anthropometrics and current levels of sports activity, offering a potential solution to inadequate graft size by selecting alternative autograft options if necessary [56,58].

An important consideration when selecting a hamstring tendon (HT) for ACLR is the risk of re-rupture and revision due to infection. Some studies have indicated that hamstring tendons are more susceptible to bacterial colonization compared to other graft options, which can lead to both low-grade and deep, high-grade infections. One proposed solution in the literature is to presoak the graft in Vancomycin before placing it into the tunnels [59]. For revision ACLR, the same autograft options are available, including the possibility of using an allograft. Although high-powered studies identifying the optimal graft choice for revisions are lacking, a systematic review by Vivekanantha et al. found that while the HT autograft remains a viable option with good patient-reported outcomes, it shows similar or slightly inferior results in terms of instability and re-rupture rates when compared to BTB and QT grafts. Additionally, HT autografts were associated with quicker return-to-play (RTP) times compared to allografts, though other benefits were not observed [56]. Although the HT autograft remains a viable option for ACLR, it is important to consider its potential shortcomings to prevent any undesirable outcomes.

### 2.3. Quadriceps Tendon Autograft

The quadriceps tendon (QT) autograft has gained attention as an alternative to other graft options, particularly due to the limitations associated with HT and bone-patellar tendon-bone BTB autografts. Initially introduced by Marshall et al. in 1979, the QT graft was proposed for ACLR using an all-soft-tissue graft extending 5–6 cm proximal to the patella and incorporating prepatellar retinacular tissue [60]. This technique was further refined by Fulkerson et al. in 1995, leading to its increased consideration as a viable graft option [60].

Despite its promise, the initial use of QT autografts faced challenges due to postoperative complications such as increased anterior laxity, extensor mechanism weakness, and a positive pivot shift in about 20% of cases. Additionally, the harvesting process, which involved the removal of a substantial portion of the QT along with parts of the prepatellar retinaculum and patellar tendon, led to significant soft tissue damage and morbidity, limiting its early adoption [41,60]. However, advancements in minimally invasive harvesting techniques and improvements in surgical procedures have sparked a resurgence of interest in QT autografts in both clinical practice and research [61,62].

The QT is the common tendon of the quadriceps muscle group, with its most superficial fibers originating from the rectus femoris and the deepest layer from the vastus intermedius. The intermediate layer is composed of fibers from the vastus lateralis and vastus medialis. While the QT has been described as bi-laminar, tri-laminar (the most common), or even four-layered, recent studies have revealed an intricate orientation of the tendon at its patellar insertion [63,64]. One of the advantages of the QT autograft is its versatility, as it can be harvested with a patellar bone plug (QT-B) or without one (QT-S). Graft-to-bone integration is critical for optimal healing, with bone-to-bone healing traditionally considered stronger and faster than tendon-to-bone healing, although a recent in vivo study has challenged this view [7,10,65]. While the QT-B harvest may carry a higher risk of patellar fracture compared to the QT-S, the latter is particularly useful in cases involving open physis. A recent systematic review by Meena et al. concluded that both QT-B and QT-S grafts are safe and effective for primary ACLR, with comparable clinical outcomes, complications, and revision rates [66].

The QT graft has garnered attention for its superior biomechanical properties. Cadaveric studies have shown that the QT graft has nearly double the cross-sectional area (CSA) compared to BTB and HT grafts, more closely approximating the native ACL’s size [7,64]. While no studies have yet determined whether this increased CSA affects the rate of QT graft impingement, the QT graft does demonstrate an ultimate load-to-failure that is higher than both BTB and HT grafts. It also exhibits greater stiffness than BTB but less than HT, which tends to have the highest supraphysiologic stiffness [7,64]. Additionally, QT grafts contain approximately 20% more collagen than BTB grafts, contributing to their increased tensile strength and stiffness, as well as a higher density of fibroblasts, which may enhance the graft’s overall strength and healing potential [7,64].

Graft fixation methods for ACLR remain a topic of debate, with no clear consensus on the best approach, as each method offers distinct advantages and disadvantages [7]. Postoperative patient satisfaction is a key outcome in ACLR, and while extensive data exists comparing BTB and HT grafts, there is relatively limited information on QT grafts. A recent systematic review and meta-analysis of six retrospective observational studies comparing QT and BTB for primary ACLR found no significant differences in postoperative function, stability, or complication rates at two years postoperatively [67]. Similar findings were reported by Meena et al., who found that both QT-B and QT-S grafts yielded satisfactory patient-reported outcomes, including Lysholm scores, IKDC scores, KOOS, and Tegner activity levels [68]. However, QT grafts have been associated with potentially lower rates of anterior knee pain and donor site morbidity compared to other grafts [8,38,68].

Runer et al. identified only two randomized controlled trials (RCTs) comparing the clinical outcomes of BTB and QT grafts, with no statistically significant differences found in any patient-reported outcomes (PROs) at two years postoperatively [8,33]. When comparing QT and HT grafts, studies have shown similar or better knee laxity measurements and patient-reported outcomes with QT grafts, along with less of a flexor muscle strength deficit, although HT grafts may have lower donor site morbidity [38]. A 2023 systematic review and meta-analysis that included nine studies on ACLR revision (RACLR) comparing QT, BPTB, and HT grafts found no significant differences in IKDC scores, Lysholm scores, VAS scores, knee laxity, return to sport, donor site morbidity, or failure rates. The QT graft used in RACLR had an overall failure rate of 7.6%, comparable to those of HT and BPTB grafts [1]. Although long-term data on QT grafts is still limited, current evidence suggests they can provide good to excellent patient outcomes, with potential advantages in certain areas. Future research should focus on optimizing graft harvest techniques, exploring long-term outcomes, and identifying patient populations that may benefit most from this graft option.

A 2021 systematic review and meta-analysis comparing QT ACLR with other grafts, including HT, BTB, QT allograft, and tibialis anterior allograft, found varied outcomes. The key finding was that early (5–8 months) post-QT ACL reconstruction, knee extensor muscle strength was not significantly different from BTB but was reduced compared to HT. Beyond eight months postoperatively, knee extensor strength following QT ACLR was comparable to that of other grafts [8,69]. The review also noted a trend toward a significantly higher isokinetic hamstring/quadriceps ratio in QT compared to HT grafts. However, knee extensor strength limb symmetry index (LSI) following QT ACLR did not reach 90%, even at 24 months postoperatively [8,69], indicating that new rehabilitation strategies may be needed to restore quadriceps strength earlier.

Return to sport (RTS) following ACLR is a common outcome measure, though it is often reported in varying ways, complicating comparisons across patient subgroups. A retrospective study of 291 young, active patients with a five-year follow-up reported a 73% RTS at pre-injury levels, with a mean time of eight months to return [8,70]. In 2022, Horstmann et al. conducted a randomized controlled trial comparing ACLR with either HT or QT grafts, finding no difference in mean time to RTS at two years postoperatively [8,71]. Unfortunately, there is a lack of high-quality studies specifically comparing RTS outcomes for QT versus HT and BTB grafts, and no systematic review or meta-analysis on this topic currently exists.

Complications and donor site morbidity associated with QT grafts have been thoroughly investigated. A recent systematic review and meta-analysis by Singh et al. analyzed 55 studies (5315 ACLR cases), including studies on QT with a bone block (B-QT), all-soft-tissue QT (S-QT), and unspecified QT grafts [72]. The pooled incidence rates for major complications included contralateral ACL injury at 6.0%, postoperative meniscal issues at 5.4%, cyclops lesions at 4.8%, graft failure at 4.1%, patellar fracture at 2.2%, hardware removal at 1.7%, infection at 1.5%, and donor-site quadriceps tendon rupture at 0.7% [72]. Graft failure, a significant concern in ACLR, was found to have no statistically significant difference in risk between QT (3.9%), HT (2.5%), and BTB (2.0%) grafts in primary ACLR [72]. To date, no complications related to QT primary ACLR have been disproportionately represented in the literature compared to other graft types. Additionally, the pooled incidence rates for minor complications included anterior knee pain at 9.7%, kneeling pain at 9.5%, sensation deficits at 4.4%, loss of extension at 4.2%, donor-site tendinopathy at 3.9%, cosmetic issues at 1.8%, and hematoma at 1.5% [72].

### 2.4. Peroneus Longus Tendon Autograft

The peroneus longus tendon (PLT) presents a newer but so far reliable option in ACL reconstruction. It occurred as a relevant option for graft harvest due to its biomechanical and anatomical properties, such as tensile strength, easy harvesting, and low donor-site morbidity [73].

The PLT spans along the lateral aspect of the leg. It originates on the proximal fibula and inserts on the medial cuneiform and first metatarsal bone. It is approximately 30 cm long with the most common thickness of the graft produced between 8 and 9 cm [73,74]. In study by Hoang et al., it was noted that the four-stranded diameter of the peroneus tendon can be sufficiently large. A graft diameter that is too large can present a possible problem when the intercondylar notch is too small. The possible solution to this is to only partially harvest the PLT, usually in its anterior part [75]. The only parameters in the current literature that were related as a predictor of the PLT graft diameter were height and weight [76]. Various methods of graft harvesting and preparation were described without a clear consensus on which one should be used in routine practice [77].

A series of work has shown the reliability and relevance of the PLT as an autograft option in ACL reconstruction. In the study by Rhatomy et al., 75 patients had isolated, single-bundle PTL ACL reconstruction. Multiple scores were used for the functional evaluation and patient-reported outcomes. The single-hop test was also used as a part of the functional evaluation. Consistent improvements over time were noted without significant losses in knee kinematics and muscle strength. This was attributed to the sparing of the thigh muscles. Donor-site morbidity was minimal, since the results on AOFAS and FADI scores, being 98.93 ± 3.10 and 99.79 ± 0.59, respectively, were considered excellent. Preservation of ankle function and low donor-site morbidity in this article are attributed to the preservation of the peroneus brevis muscle, which is, according to the literature, the dominant force in ankle eversion [78]. Another study in which PLT grafts were used concluded PLT grafts to be safe and effective option in ACL reconstruction, with satisfactory patient-reported outcomes and knee stability and good ankle stability and function [79].

Most comparative studies compared PLT grafts to HT grafts as the most common graft used in ACL reconstruction. An experimental cadaveric study by Rudy et al. showed good and comparable biomechanical properties of PLT grafts in comparison to HT grafts. The mean values of tensile strength were not significantly different between PLT and HT grafts, with a trend of somewhat higher values in the PLT group [80]. Gok et al. compared 54 patients that underwent ACL reconstruction with HT grafts to 52 that had their ACL reconstructed with PLT grafts. PLT grafts had significantly larger diameters, with a mean value of 8.56 ± 0.93 mm in comparison to 7.44 ± 0.6 mm for the HT autograft. In addition, the harvesting time was significantly shorter for the PLT graft. Having the same functional outcomes, it is also worth mentioning that the donor site morbidity was significantly more pronounced problem for the HT group. At 18 months follow-up, as expected, the HT group had significantly higher thigh hypotrophy. When donor and healthy contralateral ankles were compared, although the AOFAS and FADI scores were lower for the donor ankle, there were no clinical concerns in regard to ankle joint weakness, vascular, or neurological issues. During the period of follow-up, none of the patients developed ankle joint disfunction or problems when they returned to play [81]. The study by Fu-Dong et al. was a simultaneous biomechanical and clinical study. In the biomechanical study on 16 specimens, the tensile strength of a double PLT graft was comparable with that of a quadrupled HT graft, while both had significantly bigger tensile strength than the native ACL. A total of 38 patients with simultaneous MCL grade III rupture were admitted for an ACL reconstruction and randomized into two groups: double-strand PLT and quadrupled HT. The groups were comparable by age, sex, preoperative activity levels, and comorbidities. MCL was repaired in all patients. Regarding the functional outcomes, knee stability, and activity levels, both groups had comparable results without significant differences at 6.12 and 24 months, respectively. Ankle function was measured for the PLT group. Preoperative and postoperative measurements were performed for the donor ankle and contralateral ankle. No significant differences were noted whatsoever [74].

Besides being used as a sole graft option for ACL reconstruction, few studies have also described augmenting the HT with split thickness PLT. The studies have concluded the PLT to be a great augmenting option in patients with inadequate HT graft thickness regardless of the side used (anterior or posterior) [82,83].

In a systematic review, Jinshen et al. compared the PLT to HT as an autograft option. Comparable biomechanical properties were confirmed between two options when the diameter was the same, with a trend of higher values in PLT grafts. Most of the studies described a full-thickness PTL graft harvesting, although a partial harvest of the PLT was also described. The mean diameter of the PLT autograft was 8.5 mm; the mean Lysholm score and IKDC were favored in patients who got their ACL reconstructed with a PLT graft. No difference was noted regarding the Tegner activity scores or knee laxity. Thus, the trend of having better patient-reported outcome measures was noted for patients in which PLT grafts were used. Although the risk ratio regarding donor site morbidity and paresthesias favored PLT groups, the differences were non-significant. One of the most important negative outcomes in ACL reconstruction is graft failure. There were no differences between the two graft options with regards to re-ruptures. As expected, the AOFAS and FADI scores were decreased in patients who had reconstruction with the PLT. However, contrary to previous beliefs, PLT harvesting did not seem to affect foot and ankle function and foot arches enough to be considered as clinically relevant [77].

To date, the only negative side of PLT harvesting was its potentially negative impact on ankle stability and function. Zhao et al. described the use of a split thickness PLT autograft because of potentially negative impacts of graft harvesting on the ankle function and stability. Since the anterior fibers of the PLT are longer than the posterior ones, they are more commonly used when a split thickness graft is being used. The authors concluded that split thickness PLT grafts are safe to use, with good ankle function scores and no peroneal nerve damage or tendinopathies [84]. The literature on this topic is still scarce and conflicting. Anghtong et al. followed 24 patients throughout one year. On an average follow-up of 7 months, the peak torques of eversion and inversion during isokinetic testing were significantly lower in the donor ankle compared to the contralateral ankle. It is worth considering that only 10 patients participated in this testing. Moreover, the researchers did not perform preoperative testing in contralateral ankle but only considered the postoperative one as a reference value [85]. Zhong et al. included 65 patients with ACL rupture who had their ACL reconstructed using the PLT. Three-dimensional gait analysis was performed and the knee and ankles were analyzed. At 12 months follow-up, knee functional scores were significantly better postoperatively. Knee mobility was non-significantly different when reconstructed and healthy knees were compared. The only difference noticed was between the inversion and eversion angles of the donor ankle during the support phase. The authors recommended to implement postoperative ankle exercises [86].

Thirty-one patients were included in the study by Rhahotomy et al.; the authors compared ankle eversion and first ray plantarflexion strength between the donor side and contralateral side after ACL reconstruction. No significant differences were noticed in ankle eversion strength at the donor side in comparison to the contralateral side, with mean values of 65.78 ± 7.63 and 66.96 ± 8.38, respectively. Also, no significant differences existed in ankle first ray plantarflexion strength between the two sides, with mean values of 150.64 ± 11.67 and 152.10 ± 12.16, respectively. The AOFAS and FADI scores were not significantly different between the two sides, which had excellent scores [87].

In the systematic review by Jinshen et al., on limited number of articles, the authors concluded that no clinically relevant differences exist between PLT and HT use in ACL reconstruction with regards to postoperative ankle function and stability [77].

### 2.5. Allografts

Allografts in ACL reconstruction have had the historical advantage of eliminating donor-site morbidity, reducing surgery time and perioperative pain, and eventually leading to quicker recovery [88]. Because of those advantages, the current authors started to use cryopreserved allografts in primary ACL reconstruction more than 20 years ago [89]. Nevertheless, potential disadvantages existed at that time (immunologic reactions, slower remodeling, integration, disease transmission, and increased costs) and still represent reasons for concern currently [90].

In this context, allografts continued to be used frequently as a graft choice in both primary and revision ACL surgery. In a recent registry-based study of over 16,000 ACL reconstructions, allografts were used in 42.4% of primary and 78.8% of revision cases in the United States [91]. Currently, the main limitations on the general use of allografts in ACL reconstruction are represented by storage, infection risk, healing time and postoperative management, and return to sport. The storage of allografts can be performed as fresh-frozen, freeze-dried, or cryopreserved: both fresh-frozen and freeze-dried allografts have no viable donor cells, while cryopreservation promotes angiogenesis and reduces the host’s intravascular immune response [89]. Rigorous donor screening and recent improvements in laboratory testing have made transmission of viral diseases (i.e., HIV, HBV, HCV) extremely rare [90]. On the other hand, in 2002, the current authors [92] showed that bacterial infection following allograft ACL reconstruction was significantly reduced by sterilization and that no differences in postoperative septic arthritis risk existed between autografts and allografts. Comparing different grafts, a recent study [93] showed a lower risk of infection when a BPTB autograft was intraoperatively used if compared with a hamstring autograft and all available allografts.

Early healing has represented an interesting topic for discussion between sports medicine orthopedic surgeons. It has been historically shown that healing time varies according to graft type [17]. Many surgeons prefer, especially in professional athletes, the BPTB allograft since represents the only option to guarantee bone-to-bone healing on both sides of the knee joint. In fact, Achilles tendon and quadriceps tendon allografts contain a single bone block providing bone-to-bone healing only on one side of the joint, while frequently used soft tissue allografts include the hamstrings, the tibialis anterior, the tibialis posterior, peroneal tendons, the iliotibial band, and ultimately the fascia lata. Interestingly, a recent study by Lansdown et al. [94] showed that non-looped tibialis allografts have the lowest and quadriceps tendon grafts have the highest load-to-failure. Grassi et al. [95] studied the influence of donor age on graft tensile properties, reporting that donor age did not negatively influence the biomechanical properties of the allografts used in ACL reconstruction.

For many years, differences in postoperative management and return-to-sport time between autografts and allografts have guided graft selection. A milestone study was the one by Barrett et al. [10] [96], who showed that choosing an allograft BPTB allowed for a quicker return to sporting activities, but patients ultimately experienced increased laxity and higher incidence of failure. Because of these findings, allografts have been historically reserved for a less active population that more commonly returns to sport after a relatively long period of rehabilitation. A recent systematic review and meta-analysis by Cruz Jr et al. [97] concluded that allograft ACL reconstruction in pediatric and adolescent patients should not represent the ideal treatment option, since many studies have shown a significantly higher failure rate for allograft compared with autograft ACL reconstruction in this patient population.

Looking at clinical outcomes, Hulet et al. [90], in their recent narrative review, reported a failure rate for allograft ACL reconstruction of up to 35%. In the last 25 years, multiple systematic reviews and meta-analyses have been published on ACL reconstruction clinical outcomes, with the goal of establishing the superiority of a determined graft choice. Recently, a few authors [90,98], comparing the clinical outcome of autografts, non-chemically treated or irradiated allografts, and chemically treated or irradiated allografts, have concluded that all allograft groups had higher failure rates than autografts, though non-irradiated and non-cleansed grafts were better than irradiated and treated grafts.

### 2.6. Xenografts, Synthetics, and Scaffolds

The relatively high incidence of ACL ruptures and scientific advancements have expanded the range of options in ACL reconstruction beyond traditional autografts and allografts, incorporating xenografts, synthetic materials, and bioengineered products such as scaffolds [99,100,101].

Synthetic grafts were introduced as an alternative to bypass the possible downsides that go with auto- and allografts. Synthetic materials carry superior tensile strength at the time of fixation but can degrade over time [102]. The initial synthetic materials used were Dacron, Kevlar, and Carbon; however, they failed because of inadequate biomechanical properties and material fatigue, which led to a chronic inflammatory process. Consequently, newer synthetic materials were developed such as the Ligament Advanced Reinforcement System (LARS) and Neoligaments [103,104,105]. However, the number of research projects is still too small to recommend their routine use in ACL treatment. In addition, longer follow-up studies have shown somewhat more suboptimal outcomes than expected [104].

Xenografts have emerged as an additional alternative to previously described auto- and allografts. Most commonly derivates of a porcine/bovine source, they offer some advantages but do not come without challenges that are yet to be overcome [99,102]. They have theoretical potential to mimic the structure and properties of human ligaments. On the other hand, they are considered to be a foreign body and are prone to immunoreaction and consequent degradation with diminished performance. A possible solution for this is preprocessing, which includes decellularization, the removal of the α-Gal epitope, crosslinking, sterilization by irradiation, and adequate storage in the cold. The main goal of current research is to balance the decellularization process and native collagen preservation to maintain tensile strength. This process has been shown to be beneficial for improving immunocompatibility, while at the same time preserving biomechanical properties [106]. Most of research to date has been performed in animal models [102,106]. Clinical research is scarce in number and with heterogenous methodology. A case series with three patients by Zaffagnini et al. has shown satisfactory clinical outcomes in amateur sportsmen [99]. A study by Van der Merwe et al. included 61 patients, with 32 patients in an allograft group and 29 in a xenograft group. The groups were evaluated at 12-month and 24-month time points. Six patients in the xenograft group became infected due to a contaminated graft. After removing the contaminated and missing patients from the analysis, the results between groups were comparable. However, research suggests additional harvesting and processing improvements before thinking about the usage of these grafts in knee reconstructions [100]. Stone et al. published a 20-year follow-up of four patients who had their ACL reconstructed using a Z-lig device. All patients remained with satisfactory outcomes and fully athletic knees at this time point [107].

Tissue-engineered scaffolds represent a cutting-edge approach that combines biological and synthetic elements in aiding tissue regeneration. They are mostly used as a graft augmentation option or standalone options for ligamentous repair [103,108]. A very limited number of studies on bridge-enhancement scaffolding exist, but they show somewhat promising results in comparison to reconstruction at 2-year follow-up [101]. Bio-scaffolding ligamentous repair has also shown promising results in animal models in regard to post-reconstruction osteoarthritis onset with a biomechanically similar ligament to the ACL graft [109].

Although these unconventional methods provide some theoretical advantages to the currently standardized methods in ACL treatment, it is still far too early to implement them into routine practice of orthopedic surgeons worldwide.

## 3. Discussion: Current Trends and Controversies

In the last few decades, the popularity of the BTB graft has been challenged by alternative graft options, particularly HT and QT autografts. Hamstring grafts are frequently preferred for their potential to reduce donor site morbidity while providing comparable clinical outcomes. However, proponents of BTB grafts argue that its superior fixation strength and graft stability make it the optimal choice, especially for athletes involved in high-demand sports [2,110].

The histological and ultrastructural characteristics of the ACL are distinct from those of the tendons commonly used as grafts in ACL reconstruction, with different graft options showing considerable variability in their histological properties. Even among semitendinosus and gracilis tendons, notable differences exist in fibroblast density, blood vessel distribution, and the fibril-to-interstitium ratio [111]. A positive aspect of ACL reconstruction is that most grafts used initially possess biomechanical properties that are superior to the native ACL [112]. Although a cadaveric study on ultimate load-to-failure did not demonstrate clear superiority among the three most commonly used autografts, the quadriceps tendon (QT) exhibited more favorable structural properties [113]. Graft stiffness, an essential factor especially in the first two years post-surgery before ligamentization occurs, has been shown to be more favorable in BTB and QT grafts compared to HT grafts, which can be significantly stiffer than the native ACL [113].

For revision ACLR, autografts have demonstrated superior outcomes compared to allografts [114]. Several systematic reviews have found no significant differences among autografts, suggesting that graft choice should be based on the type previously used, the surgeon’s preferred technique, or the potential for bone tunnel enlargement [1,68,114]. A recent systematic review indicated that HT grafts produce similar or inferior outcomes in revision ACLR when compared to BTB or QT grafts [9]. A smaller study also concluded that hamstring grafts had a higher tendency for failure [115]. In pediatric and adolescent populations, revision ACLR is sometimes performed using BTB grafts [116].

In terms of infection rates following ACL reconstruction, HT autografts have been associated with a higher risk of deep infections compared to BTB autografts [117]. Similar findings have been reported in studies comparing allografts and BTB autografts, with allografts showing a greater likelihood of postoperative infection [118].

Looking into the future, xenografts or tissue engineering may offer promising avenues for developing grafts with favorable biomechanical properties without the associated donor site morbidity [107]. Additionally, there has been growing attention on the environmental impact of surgeries, particularly concerning plastic waste and the carbon footprint. A recent study revealed that extensor mechanism grafts, such as BTB and QT, have the highest carbon footprint among graft options [119].

## 4. Conclusions

Graft selection for ACL reconstruction is a multifaceted decision that requires careful consideration of both the biomechanical factors and patient-specific needs, including daily activity levels, athletic goals, and personal expectations. Advances in surgical techniques, such as meticulous anatomic tunnel placement and improved fixation methods, have contributed significantly to the overall success of ACL reconstructions. While the surgeon’s experience and preference play a critical role in graft choice, it is imperative to avoid the use of allografts in pediatric and adolescent patients due to the heightened risk of complications. There are some other clear recommendations when choosing the graft, most of them being sports specific. In sports that require sprinting or great hamstring strength (e.g., wrestling), HT grafts are not advised. On the other hand, sports that require kneeling make BTB contraindicated. The quadriceps tendon might be a wise choice for these elite athletes, a choice that combines the positive qualities of both BTB and hamstring grafts.

Ultimately, individualized graft selection, guided by a thorough understanding of the patient’s unique requirements, remains essential to achieving optimal outcomes in ACL reconstruction.

## Data Availability

No new data were created or analyzed for this study. All information discussed is derived from previously published sources, and data sharing is therefore not applicable.
